# Russian imperial borderlands, Georgian Jews, and the struggle for ‘justice’ and ‘legality’: blood libel in Kutaisi, 1878–80

**DOI:** 10.1080/02634937.2024.2302581

**Published:** 2024-02-09

**Authors:** Stefan B. Kirmse

**Affiliations:** Leibniz-Zentrum Moderner Orient (ZMO), Berlin, Germany

**Keywords:** Russian imperial law, Kutaisi trial, blood libel, antisemitism, Georgian Jews, Russian colonialism

## Abstract

This article analyses the Kutaisi Trial (1878–80), a little-known case of blood libel in the Caucasus, in which nine Jewish men stood accused of involvement in the killing of a Georgian girl. All defendants were acquitted. While the accusation of killing for allegedly Jewish ritual purposes was not pressed explicitly by the prosecution, the case was widely discussed in terms of blood libel not only by the jurists but also by the authorities, the Georgian villagers, and the press. Existing scholarship on blood libel in Russia has stressed the influence of the Russian administration over court cases and in stirring up intercultural hatred. This article, however, shows much diversity among local and central, administrative and legal actors, and paints a more complex picture of Russian imperial courts and colonialism. It is based on an analysis of archival records from Tbilisi and Kutaisi, published court transcripts, and local and regional newspapers.

## Introduction

This article analyses the Kutaisi Trial, a little-known case of blood libel in the Caucasus, in which nine Jewish men stood accused of involvement in the killing of a Georgian girl. In so doing, it explores the notions of justice (*spravedlivost’*) and legality (*zakonnost’*) in late imperial Russia. The case was heard at the Kutaisi Circuit Court in 1879 which, after a ten-day trial, acquitted all defendants. While the accusation of killing for allegedly Jewish ritual purposes was not pressed explicitly by the prosecution, everyone in the court room knew that the case was about blood libel: it was widely discussed in those terms not only by jurists but also by the authorities, the Georgian villagers and the press.

How did this accusation come about in a part of the Caucasus where Jews, Christians and Muslims had lived in close proximity for centuries? What role did the Russian imperial administration play, and how much attention did the case attract? Crucially, why did it result in an acquittal? While existing scholarship on blood libel in Russia has tended to stress the authorities’ influence over the judiciary and the role of the Russian administration in stirring up intercultural hatred, this article paints a more complex picture. Tragic accidents, such as the death of a young girl, could indeed be manipulated by state actors in the 1870s, when antisemitism was becoming ever more virulent across the empire. Yet, the accused took up the fight in the recently reformed court system and came to be aided by liberal jurists.

This article is based on an analysis of archival records from Tbilisi and Kutaisi, reports by the local authorities, and the coverage of the case in Tiflis-based and Jewish newspapers.^1^

## Kutaisi and the history of blood libel in the Russian Empire

Like most, if not all, accusations of ritual murder, the Kutaisi case was not primarily about the act of killing; it was about the fact that the purported criminals and their actions were framed as religiously and culturally motivated. As in the overwhelming majority of such cases, the accused were Jewish.^2^ Few people truly believed that the defendants in the Kutaisi case had abducted and killed the girl in question to use her blood for religious rites. However, when the authorities realized that this belief, mixed with anger and despair, was entrenched among local Christians and was causing rampant antisemitic attacks, they decided to push for an open trial in order to defuse the conflict. In so doing, they succeeded and failed at the same time: while the trial cleared the names of the accused, the attacks on these individuals and the Jewish community as a whole did not stop. A young girl had died and someone had to pay. Thus, the case is about a variety of related issues: antisemitism, interreligious relations and conflict in the South Caucasian countryside, and the ways in which the imperial judiciary and state administration tried to handle such cases.

This article seeks to make sense of both the legal strategies pursued and the final outcomes by tracing the diversity and development of ideas of justice (*spravedlivost’*) and legality (*zakonnost’*) as expressed in the courtroom. It further sets out to document the ways in which the key actors—the prosecution and authorities, the accused and their defenders—used these ideas and the legal language they produced as practical tools to impress the judges and win their cases.

The Kutaisi case reveals different levels of ‘justice’. After the Russian Empire’s Judicial Reform (1864), judges only had to follow their conscience, and thus the operation of the courts ensured the coexistence of different notions of justice (*spravedlivost’*), that is, justice as a flexible moral compass. In addition, justice became a rhetorical and practical instrument that enabled lawyers, judges, and simple litigants to fight for a desired outcome. The same was true of legality/lawfulness (*zakonnost’*), the question of whether a decision or process was legally sound. In the case at hand, moreover, rhetorical strategies did not remain limited to calls for individual justice. Since the case was about the ‘culture’ of a religious minority, they invariably touched on broader emancipatory debates and struggles—along with memories of historical injury. Thus, justice also emerged at the level of ideological projects, not unlike the calls of reformers and revolutionaries for ‘social justice’ in the second half of the nineteenth century.^3^

Kutaisi is part of a longer history of blood accusations against Jews in the Russian Empire. As Klier (1986) documented, such accusations arrived in the empire from East-Central Europe, with its large Jewish population, only in the late eighteenth century. By the mid-nineteenth century, the subject was still only beginning to make a mark on Russian public discourse. Previous cases of anti-Jewish blood libel—Senno/Belarussian province in 1799 (Klier 1986, 18–19), Velizh/Smolensk province from 1823 to 1835 (Avrutin 2018), and Saratov on the lower Volga from 1853 to 1860 (Reed 2017)—differed from Kutaisi in several respects. All took place before the emergence of an independent judiciary, created by the Judicial Reform, which meant that they were handled by a mixture of local and central executive authorities, including the Interior Ministry. Jurists only played secondary roles in these cases, as they were only beginning to coalesce a professional group. Further, before the Great Reforms of the 1860s, there were no public trials or adversarial court procedures on a large scale across the empire. The ‘public sphere’ and its new institutions for the masses, including schools, universities, theatres, and mass publications, was still in its infancy. Thus, there existed neither the institutions nor the public discourse that would accompany later cases of blood libel. It was only outside the Russian Empire that mid-nineteenth century cases would generate a broad public response, as in the Damascus Affair of 1840 (Frankel 1997).

Perhaps the only ‘modern’ case of blood libel that ever occurred in tsarist Russia was the Beilis Affair in Kiev (1911–13). In the context of ritual murder trials, two elements have been associated with ‘modernity’ (largely absent in medieval and early modern cases): transnational mobilization, and the reliance on forensic, medical, psychological and other ‘expertise’, rather than cultural prejudice and (false) testimony (Avrutin, Dekel-Chen, and Weinberg 2017; Kieval 2022, esp. 135–138). The trial of the Jewish office clerk Mendel Beilis, accused of killing and draining the blood of a Christian boy, became a truly international affair and press sensation (Dekel-Chen 2017; Kotik 1978; Levin 2014; Weinberg 2014). Contemporary observers dubbed it the Russian Dreyfus Affair.^4^ Those supporting Beilis mobilized international networks and started campaigns to gain public support, whereas those charging Beilis would argue in a typical antisemitic manner how international financial networks were trying to obstruct justice. In other words, the ‘tools of modernity’, as Avrutin, Dekel-Chen, and Weinberg (2017, 12) put it, were invoked and utilized by all sides in 1913 far more than in previous cases.

Science and pseudo-science were also crucial to ‘modern’ ritual murder accusations, without which they might have never become as big as they were. In his analysis of the (non-Jewish) ritual murder trials surrounding the Multan case of 1892,^5^ Geraci shows how ethnographers endlessly testified in court to the existence of blood rituals among the Votiak people in the Volga-Kama region (Geraci 2002, 195–222); and following the advent of ‘race science’, which downplayed the religious element in the blood accusation in favour of more secularized charges, psychiatrists were among the driving forces behind the case against Beilis (Hillis 2013, 244–273; Mogilner 2013, 167–184; 2017). By comparison, in the Kutaisi and other, earlier cases, experts were consulted only to a degree, and mostly indirectly.

Still, in many ways, Kutaisi was a far cry from earlier cases, not only elsewhere in the Russian Empire, but also in Georgia, where it was not the first instance of blood libel. The Surami case, which occurred in central Georgia in 1851–53, unfolded less than forty miles from the scene of the crime in 1878. Its trajectory, however, reflected the pre-reform judiciary and administration. In Surami, seven Jewish individuals, all learned men versed in religious matters, were falsely accused of the murder of a Christian (Georgian) boy for religious purposes. While there was no evidence against them, local investigators pressed the case for three years before the Senate in St Petersburg ultimately made a decision, convicted and exiled the accused to Siberia, each to a separate, remote place where they were to be kept under ‘strict supervision’ (Ben-Oren 1984, 73). Soviet, Israeli and Georgian scholars agree that the Russian imperial state, particularly Viceroy Mikhail Vorontsov and his representatives and police investigators, were heavily involved (Ben-Oren 1984), even manipulated the case to ensure a conviction (Mamistvalishvili 2014; Shukyan 1940).^6^ For Mamistvalishvili, antisemitism only arrived in Georgia with the Russian army, and for the ‘backward masses’ it then became hard to distinguish truth from fiction (Mamistvalishvili 2014, 210–211). This reflects a broader claim in modern Georgian historiography and politics: that Georgians and Jews had lived peacefully together in the South Caucasus for over two millennia.^7^

Gershon Ben-Oren’s historical investigation highlights another two points relevant as background for the Kutaisi case. First, even without major press coverage, Surami developed an international dimension insofar as local rabbis informed their brethren in Constantinople, who then wrote to the philanthropist Moses Montefiore in London. The latter began to correspond with Prince Vorontsov via the Russian embassy.^8^ Ultimately, though, the geographical reach of the case remained limited: Montefiore achieved very little, and his efforts were not even widely publicized before 1871 (when the contents of his correspondence with Vorontsov were published in Odessa and Tiflis^9^). Second, and more importantly, the Surami accusation stuck, for the rumours and sporadic violence against the Jewish population persisted. In 1869, the Hebrew-language weekly *Ha-Magid* (The Narrator) was still reporting about Surami Jews complaining to a visitor from the Paris-based *Alliance Israélite Universelle*: ‘The cruel Armenians and Georgians […] always perpetrate the blood libel against us […] and wait for the right time to carry out their evil plans to attack our houses [ … ].’^10^ Riots against Jews indeed shook Kutaisi, Tiflis and Surami in the 1870s (Ben-Oren 1984, 74; Meskhi 1877). In fact, as we will see below, there is a direct link between renewed rumours of blood sacrifice and antisemitic attacks in Surami in 1877 and the beginning of the Kutaisi case the following year.

Scholars of the blood accusation in the Russian Empire, including the aforementioned works on Velizh, Saratov, Surami and Beilis, have tended to stress its political dimension. They have shown how government officials and Russian conservatives tried to exploit the cases for political benefit: to justify the empire’s continuing discriminatory laws and regulations against the Jewish population, to highlight Russia’s civilizing mission toward its internal ‘Others’, and in some cases to curb the growing revolutionary movement (as Jews came to be perceived and framed as left-wing revolutionaries).^11^ That said, most agree that the Russian government never had any grand plans and simply tried to exploit emerging developments. As Rogger pithily put it, it was less the political establishment that pushed for the Beilis trial than ‘a small band of unsuccessful politicians and honest maniacs’ (Rogger 1966, 629). Geraci similarly highlights the ways in which the Multan case became politicized and offers a vantage point for discussing Russian imperial governance (Geraci 2002, 195–222).

Finally, several scholars have analysed the discourses facilitating and sustaining late imperial blood libel. Katsis (1995) and Weinberg (2017) have addressed the broader intellectual discourse surrounding the Beilis case, rooted in the Silver Age culture, Russia’s own fin-de-siècle cultural turn, with its growing interest in religion, mysticism and secret rituals. Mogilner (2017) has focused on the contemporary international discourse about race and imperial superiority to explain the development of Multan and Beilis. And perhaps closest to my own approach, Murav (2000) has shown how key legal players—the prosecution and defence in the Beilis case—rooted their lines of argumentation in different sets of discourses: religious mysticism versus *zakonnost’*/legality and the sober discussion of facts. Her discussion of Beilis is one of the rare analyses of blood libel in Russia that gives full attention to the complicated role of the judiciary.

And what about specific literature on the Kutaisi case? Surprisingly, perhaps, there has been little scholarly discussion. The case is mentioned in passing, notably by scholars of Dostoevsky (Grossman 1934, esp. 110–113; Murav 2017, esp. 157–158), as the acquittal gave a boost to Dostoevsky’s antisemitism. Works on Surami or Jews in Georgia include but short paragraphs on Kutaisi (Ben-Oren 1984, 75; Shukyan 1940, 85–86). This is striking insofar as the full transcript of the trial was published in Russian as early as 1879.^12^ On the occasion of the centenary of the case in 1978, ‘*Bnei Brith*’, the organization of Georgian Jews in Israel, produced a full Hebrew and a shorter German translation of the transcript (Megrelishvili 1978a, 1978b). While these publications limited themselves to reproducing court materials and contain virtually no historical analysis, the material is framed in a way that differs significantly from the classic narrative on blood libel (namely, its use and manipulation by the authorities). In his introduction to the translations, the editor Gershon Megrelishvili stressed the importance of the new courts and the ultimate acquittal: ‘For the first time in 750 years of the rampage of blood libel against Jews, an acquittal verdict by the court was heard’ (Megrelishvili 1978a, 9). He then praised the liberal lawyers who skilfully defended the accused. This take on the case is echoed in the preface to the Hebrew edition, written by the head of the Israeli Chamber of Lawyers, who contended: ‘The Kutaisi trial, conducted only 14 years after the [Judicial] reform, illustrates the reforms’ strength. The trial also reveals the power of Russian liberalism, which fought the people’s war against tyranny [*melkhemet ha’am neged ‘aritsut*]’ (4).

This is indeed one of the reasons why the final plea made by the defence lawyer Petr Aleksandrov was later reprinted by imperial, Soviet and post-Soviet publishers, yet mainly with lawyers in mind and therefore still without historical analysis (e.g., Aleksandrov 2011).

In this article, I also highlight the power of the judiciary, along with its limits, while arguing that the Russian authorities were diverse and pursued a range of agendas. Before examining the case, however, let me add a factor in Kutaisi that has received little attention in discussions of blood libel in the Russian Empire: that neither the accused nor the injured party were Russian or spoke much Russian. This small detail delegated the imperial authorities the role of an external mediator. In his discussion of the Surami case, Mamistvalishvili argues that such mediation was a fiction. In fact, framing Russia’s role in such terms was little more than a hypocritical justification of colonialism: the administration wanted to create the impression that Russian rule was protecting the Jews of Georgia from the antisemitism of the local population, whereas it was actually that very administration that posed the greatest danger to the Jewish population (Mamistvalishvili 2014, 214). The analysis below, however, will suggest that this is too simple.

### The death of Sarra Modebadze

**Figure 1.**
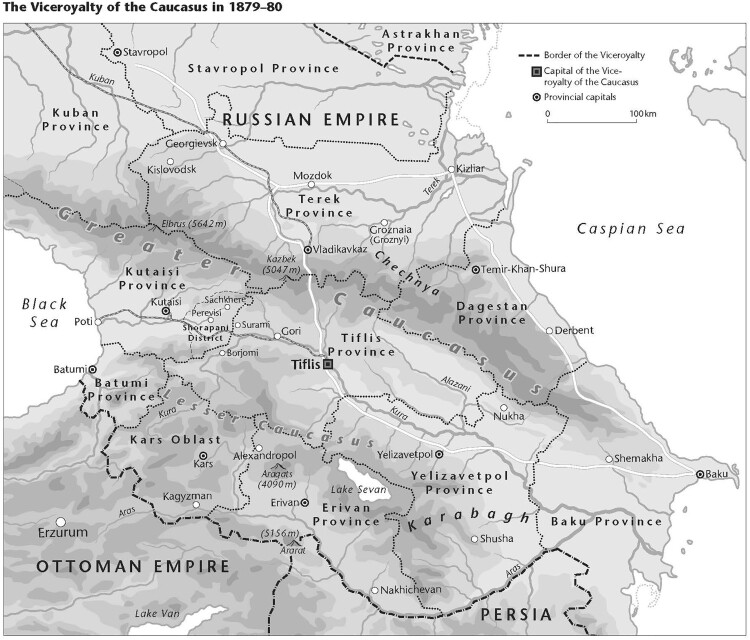
Viceroyalty of the Caucasus with key locations for the case, 1879–80. *Source:* © Peter Palm, Berlin.

The Kutaisi case began in early April 1878 in the eastern part of Kutaisi province, centrally located in the Viceroyalty of the Caucasus (*Kavkazkoe namestnichestvo*) (Figure 1). Indeed, the viceroy and the head of his administration would take on key roles in this criminal case. As in the rest of Georgia, a small Jewish minority—less than 1% of the population—had been living in this region for a long time. They were well-integrated ‘Georgian Jews’ (*kartveli ebraelebi*), that is, people whose mother tongue was Georgian and who therefore differed from the Russian- or Yiddish-speaking Ashkenazi Jews in Tiflis and other big cities (Gachechiladze 2021). By the 1870s, this community had been affected by at least two key developments. First, the Emancipation Reform, implemented in Kutaisi province from 1865, had left the bulk of the Georgian Jews, many of whom had previously been enserfed, without land, which drove them into livelihoods as trader–peddlers; and second, rapid improvements in infrastructure and trade had spurred the formation of a new class of affluent, well-connected Jewish merchants in towns such as Kutaisi (Mamistvalishvili 2014, esp. 263–264, 313–319).

**Figure 2.**
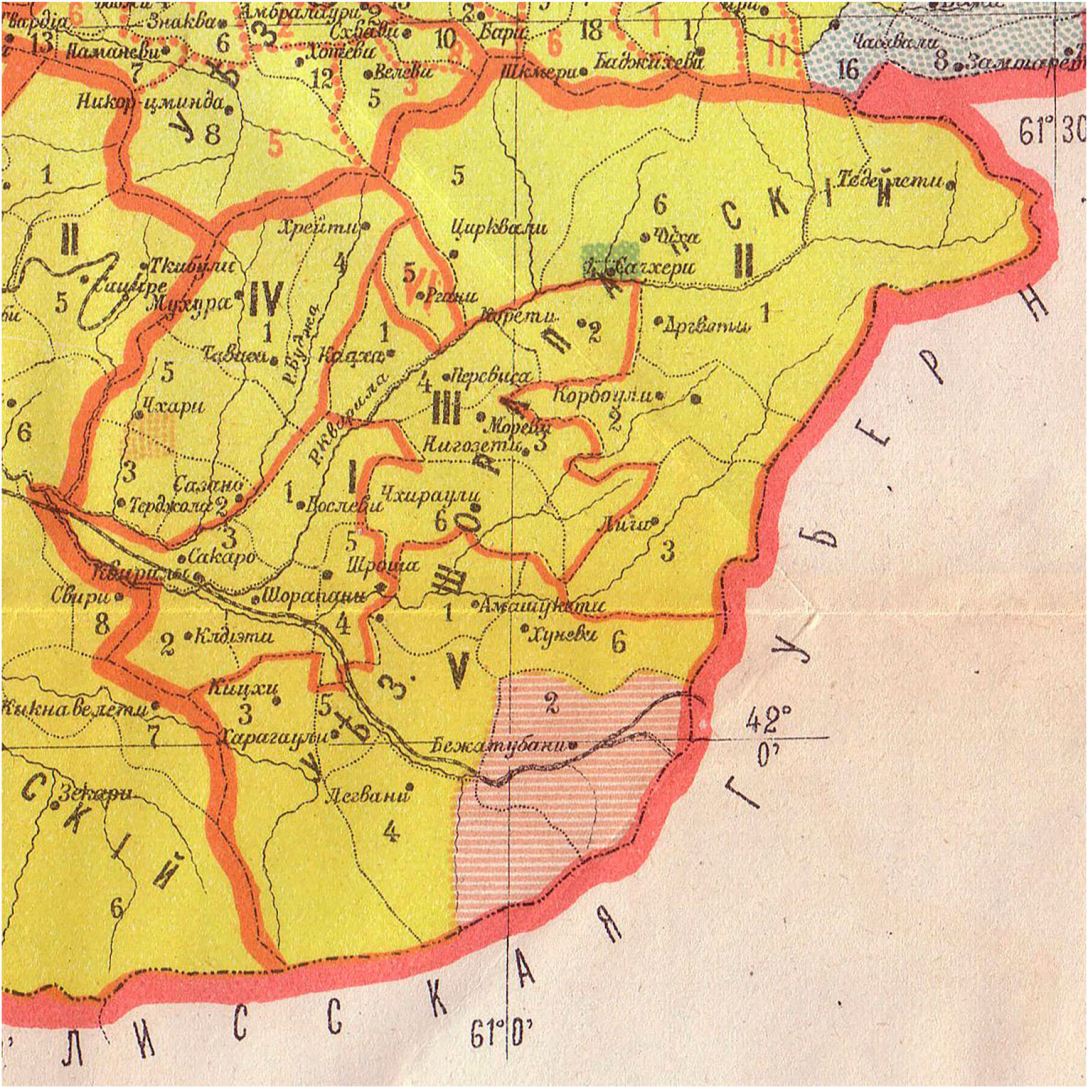
Shorapani district on the border of Tiflis province (to the east) showing the region’s ethnic composition, 1886. *Source:* https://upload.wikimedia.org/wikipedia/commons/9/94/Map-etno-kutais.jpg Note: This map uses the colour green for the Jewish population, which is concentrated in Sachkhere.

In Shorapani district, where the mysterious death took place, Georgian Jews mostly lived in the village of Sachkhere (Figure 2). A statistical survey published in Tiflis in 1880 put the number of Jews in Sachkhere at 628 people.^13^

On 6 April 1878, seven-year-old Sarra Modebadze was found dead by a stone wall. The body lay about 2.5 kilometres from her native village of Perevisi, where she had disappeared two days earlier.^14^ A preliminary court investigation discovered that on that fateful day she had left her parental home with her older sister to help their neighbours make white lead (for use in makeup). This happened in a small grove, off the Sadzalikhovskaia Road, which led through the village of Perevisi and on to Sachkhere. At about three p.m., Sarra left the group unnoticed, presumably in the direction of her parents’ house. She never arrived. Her family soon went out to look for her, and helped by many neighbours, they continued their search all night and on the following day.

The girl’s father, Iosif Modebadze, quickly reported the disappearance to the local elder Niko Darbaidze; and in so doing, he expressed the suspicion, purportedly shared by other villagers, that Sachkhere Jews had kidnapped his daughter.^15^ Darbaidze then asked the village school teacher Mikadze to draw up a formal police notification and sent it to the police captain, Prince Abashidze, in Sachkhere.^16^ In court, Sarra’s father later explained:

When I learnt that Yids [*uriebi*] had been in our village the day before, I wondered if they had taken the child. I had heard that Yids needed Christian blood and remembered that something similar had happened in Surami. That’s why I gave up the search and went to Sachkhere to tell the police captain.^17^

After the body was discovered, Darbaidze quickly notified the police that the dead girl showed deep cuts with missing skin on both hands, obvious allusions to blood rituals.^18^ The report of a crime to the police, in turn, set the judiciary in motion, as was required by the Statutes of Criminal Court Procedure, extended to the South Caucasus in 1867, which put the Kutaisi Circuit Court investigator on the job.^19^

The cause of death was not entirely clear. The Shorapani district doctor, who carried out an autopsy on 27 April, argued that the girl had perished in a tragic accident: she had drowned during a downpour.^20^ This echoed the initial reasoning of the court investigator from 13 April that heavy rain had caused strong currents on the road.^21^ The conclusion, however, seemed strange to many witnesses, who claimed that it had been dry on the days in question. Furthermore, the girl’s body and clothes showed little sign of exposure to water. Thus, a second autopsy was conducted by the Kutaisi province doctor on 16 May, who argued that Sarra Modebadze had suffocated.^22^ Asked to assess these two differing conclusions, the Medical Department of the Civil Administration of the Caucasus eventually explained that only one thing was clear: the girl’s death had been caused by a lack of air in her lungs.^23^ This could have been the result of drowning or suffocation, but it was impossible to say for sure. To make matters more complicated, there were no signs of violence on her body other than cuts on her hands, which according to the district doctor had been inflicted posthumously, probably by small animals.^24^ This assessment, however, would not stop the prosecution from later arguing in court that the lacerations looked ‘as if they had been inflicted by a tearing instrument’.^25^ The initial police investigation also observed that the victim’s ‘palms and soles were astonishingly pale’, thus indirectly making a case for blood loss.^26^

The family and their community were understandably inconsolable. They would not accept a possible accident as the cause of death, especially since the girl could have walked off in a direction where two groups of Jewish men had been seen, headed for Sachkhere, around the same time. The men were difficult not to notice because they were making a lot of noise, carrying chickens, geese, and a little goat on their horses. A more ominous voice was also heard: a child’s voice, according to at least two witnesses. And thus, to the villagers and the local authorities, the case seemed clear: the Jewish men had first abducted the girl and later dumped the body after it was no longer of any use.

Ten days after the girl’s tragic death, violent antisemitic attacks started shaking the district. In late May, on behalf of the Jewish community of Sachkhere, two persons, Moshe Petruashvili and the local rabbi Yakov Mamistvalov, sent a petition to the tsar’s representative (and brother) in Tiflis, Viceroy Prince Mikhail Nikolaevich, informing him that, on 14 April, their Christian neighbours, ‘mostly from among the simple class’, had begun to beat and rob them ‘just like the Surami Jews [the previous year]’.^27^ The petitioners praised the police captain Abashidze for maintaining order with his Cossacks that day but added that ever since then, Jews continued to be chased, robbed, beaten up and even stabbed at every possible encounter.^28^ The trigger for these attacks, the petition went on to explain, had been the ‘superstitious defamation’ (*suevernyi izvet*), spread by Sarra Modebadze’s father and others, that Jews had abducted the girl to obtain her blood for Jewish rituals.^29^ The petitioners’ account is confirmed by correspondence between the Kutaisi Circuit Court and the organ to which it reported, the Tiflis Judicial Chamber: the victim’s parents suspected the Jews because their daughter’s disappearance coincided with the Jewish Passover.^30^ According to the petitioners, the family then mobilized ‘false witnesses’ (*lzhesvideteli*) while the Kutaisi Circuit Court investigator launched a criminal investigation ‘without any legal basis (*bez vsiakago zakonnago osnovaniia*)’.^31^ To please the crowds (*v ugodnost’ naroda*), he even arrested two Jewish men, who, by the time the petition was sent, were still languishing in prison.^32^ The arrests, in turn, only helped to convince people of the reality of the accusation.^33^

Indeed, while the district head first announced drowning as the cause of the girl’s death on 13 April, he soon afterwards concluded that the death had been the result of ‘premeditated murder’ (*umyshlennoe ubiistvo*).^34^ The police captain’s enquiries quickly produced the names of the seven men who had travelled through Perevisi on the day of Sarra Modebadze’s disappearance.^35^ They had crossed the village in two groups, seen and heard by numerous residents. Among the police findings, several stood out: on one of the Jewish traders’ fully laden horses, witnesses had not only spotted chickens and geese but also a large saddle bag (*peremennaia sumka*), half of which had been occupied by a young goat, crying because one of the men kept hitting it with a whip.^36^ The witness Solomeia Kolmokhelidze claimed to have heard the cry (*krik i plach*) of a child, and although another witness thought that Kolmokhelidze had only heard the young goat, the woman remained certain that it had been a child’s voice.^37^ Other witnesses insisted that they had heard a child’s cry for help and seen the Jewish men tie up one of the bags.^38^

According to local Georgian nobility, also residents of Sachkherе, the (drunk) Jewish man Mikhail Abramov Elikishvili came running into the home of the marshal of the nobility (*predvoditel’ dvorianstva*), Prince Siko Tsereteli, and shouted that he wanted to let the marshal know that Jews had recently killed a Christian child.^39^ Overhearing this, several Jewish men allegedly rushed to the scene and dragged Elikishvili off the marshal’s property.

Thus, ‘the Jews who had accompanied Sarra Modebadze on the road’, as the prosecution put it, became the prime suspects.^40^ ‘Accompanied’ was a liberal interpretation, for the victim and the accused had never been seen together. Local investigations, however, suggested that Sarra had been walking alone on the road in close proximity to the first group of (four) Jewish men, who had a large saddle bag on one of their horses, and that the second group of (three) Jewish men was not far behind.^41^ Another point that reinforced the idea of an abduction was that on the first day of the search, many people claimed to have walked past the stone wall where the body would eventually be found. Thus, the Kutaisi prosecutor Lidov reasoned, the body must have been dumped there the night before its discovery.^42^ In addition, the prosecution pointed out that neither the girl nor her family ever passed through the spot by the stone wall^43^—another argument for the ‘kill and dump’ theory.

To be fair, then, the suggestion that an abduction had taken place was not unfounded. What was far less believable was its supposed execution by the Jewish men in question. The evidence against the men from Sachkhere was all based on dubious testimonies. For the court investigator and Kutaisi prosecutor, however, it was sufficient. Drawing on the medical tests, the locations in question, and numerous testimonies, the investigator concluded on 8 June that the Sachkere Jews had ‘in all likelihood’ participated in the abduction and killing of Sarra Modebadze.^44^ He therefore ordered the arrest of two of the men, citing undefined ‘circumstantial evidence’ against them, the lack of counter-evidence, and the need to prevent collusion (*stachka*) between them and the other seven suspects, who had not been located yet.^45^ Lidov explained to the higher authority in Tiflis that he considered the court investigator’s decision to be ‘completely accurate and legal’ (*vpolne pravil’nym i zakonnym*), both in light of the evidence and because several suspects had already ‘managed to go into hiding’.^46^

Meanwhile, the local situation had got out of hand. The Kutaisi governor’s instructions to village elders, issued in mid-April to calm down the masses, had produced no palpable results, as the aforementioned petition, sent on 31 May, underlined.^47^ The Jewish community, in their own words, could no longer engage in trade, ‘meet their tax and service obligations’, or ‘make use of the protection of the law’ (*pol’zovat’sia zashchitoiu zakona*). Without trade, they could not exist, because they did not own any land. The blood accusation, the petitioners concluded, was nothing but ‘superstition, slander, and viciousness by unenlightened Christians’.^48^

They cited various points to illustrate this. First, they argued that the blood accusation was not mentioned in the criminal laws of the Russian Empire, or any other ‘enlightened European state’—and if it actually existed, then there would surely be a penalty defined for it.^49^ Second, they insisted that the historical investigation ‘On Some Medieval Accusations against the Jews’, published in 1861 by Professor Daniil A. Khvol’son (St Petersburg University), had documented the long history of the blood accusation, with ‘all enlightened jurists and professors’ coming to the conclusion that Jews did not need the blood of Christian children.

The petitioners’ acquaintance with this famous professor—who pragmatically converted from Judaism to Orthodox Christianity to take up a professorship in Hebrew in 1855—is curious. Reed’s research suggests that the professor was contacted by two petitioners from Sachkhere on 27 April 1878 and accepted the challenge to write a follow-up piece to his 1861 treatise (Reed 2014, 230). The enlarged second edition was then published in early 1880, with a circulation of 10,000 copies (Günzburg 1967, 337). In the preface, Khvol’son acknowledged the Kutaisi case as part of his motivation for writing it (Khvol’son 1880, XII). That said, the professor’s influence over the case may have been even more substantial. In the introduction to the German translation of his book, he explained that, as early as 1878, he had sought an audience with Viceroy Mikhail Nikolaevich, who happened to be in St Petersburg and whom Khvol’son described as a man with ‘an unbending love of justice’. The viceroy accepted various copies of Khvol’son’s book and ordered them to be distributed among court staff in Tiflis and Kutaisi (Khvol’son 1901, XII). In 1881, on his way back from a conference in Tiflis, the professor then passed through Kutaisi, where he met the judges of the case and visited the Jewish community, with notable local attention (Günzburg 1967, 337–338). Khvol’son later claimed that on this occasion, both the judges and the marshal of the nobility told him that his book had convinced them of the defendants’ innocence (Khvol’son 1901, XIII).

But let us return to the petition of May 1878. The petitioners raised a third point in their letter to the viceroy. They mentioned the instruction by Emperor Alexander I of March 1817 that Jews were not to be accused of the killing of Christian children without evidence; that if a murder occurred and Jews were suspected to have committed it for ritual purposes, then an investigation would be launched on legal grounds (*na zakonnom osnovanii*).^50^ The petitioners chose not to mention that the next tsar, Nicholas I (the viceroy’s father), had refused to renew this instruction after the settlement of the Velizh Affair in 1830.^51^ In conclusion, the petition asked the viceroy to put an end to their ‘persecution and torment’ by Christians, ‘by persuasion or force’, to set up a commission for an investigation into the blood accusation; and to punish those responsible for the death of Sarra Modebadze, in accordance with criminal law, along with those stirring up hatred against the Jews ‘along with their false witnesses’.^52^

In response, the viceroy’s administration insisted the Kutaisi governor take the most energetic measures to ensure the safety of the Sachkhere Jews’ and asked the Tiflis Judicial Chamber for the legal grounds upon which they had been arrested.^53^ Much time was lost by reports being sent back and forth while the situation on the ground was getting worse. On 17 June, Rabbi Mamistvalov and Petruashvili sent a second petition.^54^ This time round, they did not ask for protection from the ‘ignorant masses’, as they put it, but from the local court investigator and prosecutor.^55^ At this point, all nine suspects had been arrested.^56^ The petitioners insisted that, naturally, the law allowed the court to take suspects into custody; however, in this case, ‘extreme care’ would have been needed because the people would see the arrests as ‘a fact confirming the accusation’. They also wondered why there had been such a rush to make arrests. Was it the fear that the suspects would run? That did not make sense to them: if the accusations were true, then ‘all Jews’ (underlined in the petition) would be complicit in the crime, and these large numbers clearly would not be able to flee.^57^ The petitioners thus asked the viceroy to revoke the arrests or, at least, to release the suspects on bail.^58^

The Kutaisi governor only sent a brief statement in response to the viceroy’s request for action.^59^ He explained that the infuriation of the Christian population towards the Jews was ‘completely justified’ since ‘the investigation had confirmed the fact of the little girl’s murder by the Jews’. He also asserted that, regardless, no violence of any kind had been permitted against the Sachkhere Jews and reassured the centre that measures had been put in place to protect this population from attacks.

The viceroy’s administration was unconvinced. In July, it dispatched the prosecutor of the Tiflis Judicial Chamber, Valerian Andreev, to Kutaisi.^60^ In a telling explanatory letter, the head of the viceroy’s administration informed Andreev of political concerns surrounding the case that cast an interesting light on the viceroy’s role and position. Alluding to earlier blood accusations and violence in the region, he argued that the death of Sarra Modebadze and the subsequent arrests had led the ‘constant antagonism’ between the Christian and the Jewish populations to take on ‘an extreme degree of tension’.^61^ At the same time, his verdict on the Kutaisi court was devastating: ‘The data used by the court investigator as grounds for the arrest of several Jews [ … ] are completely unconvincing (*sovershenno ne ubeditel’nymi*).’^62^

Yet, his prime concern was political rather than judicial. While a quick release of the prisoners could be seen as ‘necessary and just’ (*nuzhna i spravedliva*), the mood among Christians could lead to ‘accusations against the authorities’ as well as to ‘popular vengeance’ against the Jews.^63^ A release could only happen in the medium-term: if it were to prove impossible to find the real culprits, the Christian population would have to be convinced that the investigation had proceeded with the utmost impartiality but had not produced any evidence against the Jewish defendants.^64^ Thus, Andreev was told to facilitate the release of the prisoners ‘if further investigations make it possible’.^65^ Interestingly, this rather neutral formulation was chosen to replace the original wording for Andreev’s task, crossed out in the manuscript, namely ‘to initiate, if need be, further investigation by a more experienced and active court investigator (*bolee opytnyi i bolee deiatel’nyi sudebnyi sledovatel’*)’.^66^ Clearly, the investigator had done a shoddy job.

The Judicial Reform of 1864 had made the Russian Empire’s judiciary—judicial chambers and circuit courts—independent of the executive and administration, with the exception of the prosecution, which still reported to the Ministry of Justice. This is the reason why the office of the viceroy could interfere so directly with the prosecutor’s work. In many ‘ordinary’ provinces, the central authorities and their local representatives had major difficulty exercising power over the judiciary at this time (Kirmse 2019). The central authorities and governors would seek to have as many ‘troublemakers’ as possible arrested while the courts routinely ignored such pressure. It is striking that in the Kutaisi case, it was the local actors—court investigator, prosecutor and governor—who stood behind the accusation, whereas the administration in Tiflis was far more hesitant, probably because of the case’s political implications, but perhaps also (if we trust Khvol’son’s opinion) because of the viceroy’s personal scepticism towards blood libel.

Andreev, however, chose to defend the work of the local institutions. There had been sufficient evidence, he asserted, that five of those in custody had indeed kidnapped Sarra Modebadze ‘for an unknown reason’.^67^ Four of the detainees, by contrast, could be set free on bail.^68^ Like the victim’s relatives, he also made reference to Passover, arguing that on the day the body was discovered (6 April), just before dawn, two witnesses ran into one of the accused, Moshe Sotsiashvili, who was carrying an empty saddle bag on the road between the stone wall and Sachkhere and asked them not to tell anyone about the encounter. Andreev added: ‘According to [Jewish] custom, no one can leave the house during that night,’ thus suggesting that Sotsiashvili had been up to no good.^69^ He ignored the possibility of false testimony. Nor did he seem worried that his reasoning was deeply contradictory, as he allowed for the possibility of ritual murder while arguing that the men had broken religious custom. Andreev proposed not dismissing the investigator because he ‘had not found any mistakes in his work nor any bias against the accused’.^70^ He also asked for the prosecutor of the Kutaisi Circuit Court to remain in charge.^71^

Overall, Andreev painted a picture of inter-religious relations in Kutaisi province that echoed the governor. The Christian population, he claimed, ‘[was] not intending to inflict any violence on the Jews’ while there was indeed an ‘alienation’ (*otchuzhdenie*) and tension that would, however, pass swiftly.^72^ There had only been three cases of Jews being robbed, which were under investigation, and these events ‘did not bear the character of an intentional persecution of Jews’.^73^

The viceroy’s response was to ask for updated bimonthly reports, thus underlining the importance of the matter.^74^ By mid-September, however, the investigation was over.^75^ Andreev suggested that Sarra Modebadze had probably died because of an abduction; at the same time, he argued that the case would not allow for an exploration of Jewish rites ‘because the abduction could have been carried out for another purpose, such as the sale of the girl, her conversion to Judaism, or similar’.^76^ While blood libel, then, was perhaps not at the centre of the accusation, cultural prejudice and antisemitism certainly were.

The head of the viceroy’s administration remained unconvinced and once again struck a more sceptical note when forwarding his assessment to Prince Mikhail Nikolaevich. He argued that it would be ‘unconditionally necessary’ to have a trial in open court. Any administrative order ‘from the top’ would only convince the Christian population that the accusations were true.^77^ The unwritten subtext was that only a public trial could dispel these ideas. The viceroy pencilled his response on the first page of the assessment: ‘Act in accordance with your opinion, which I share completely.’^78^ A trial was the obvious solution.

### The Kutaisi trial

In November 1878, it was decided that the case was to be tried in the spring, for time was needed to send court summons to sixty-eight witnesses.^79^ When the viceroy’s administration informed the Kutaisi governor in February 1879 that there were still plenty of petitions coming in from Sachkhere Jews—reporting beatings (even by police) and thefts,^80^ the governor once again responded defensively. His police, he claimed, ‘forwarded all lawful complaints (*zakonnye zhaloby*) to the appropriate institution’, refusing only cases of ‘harassment by complainants, mostly concerning payment issues with the Christian population’.^81^ Any inter-religious clashes, he asserted, if they actually occurred, had nothing to do with an allegedly hostile Christian attitude towards the Jews.^82^

**Figure 3.**
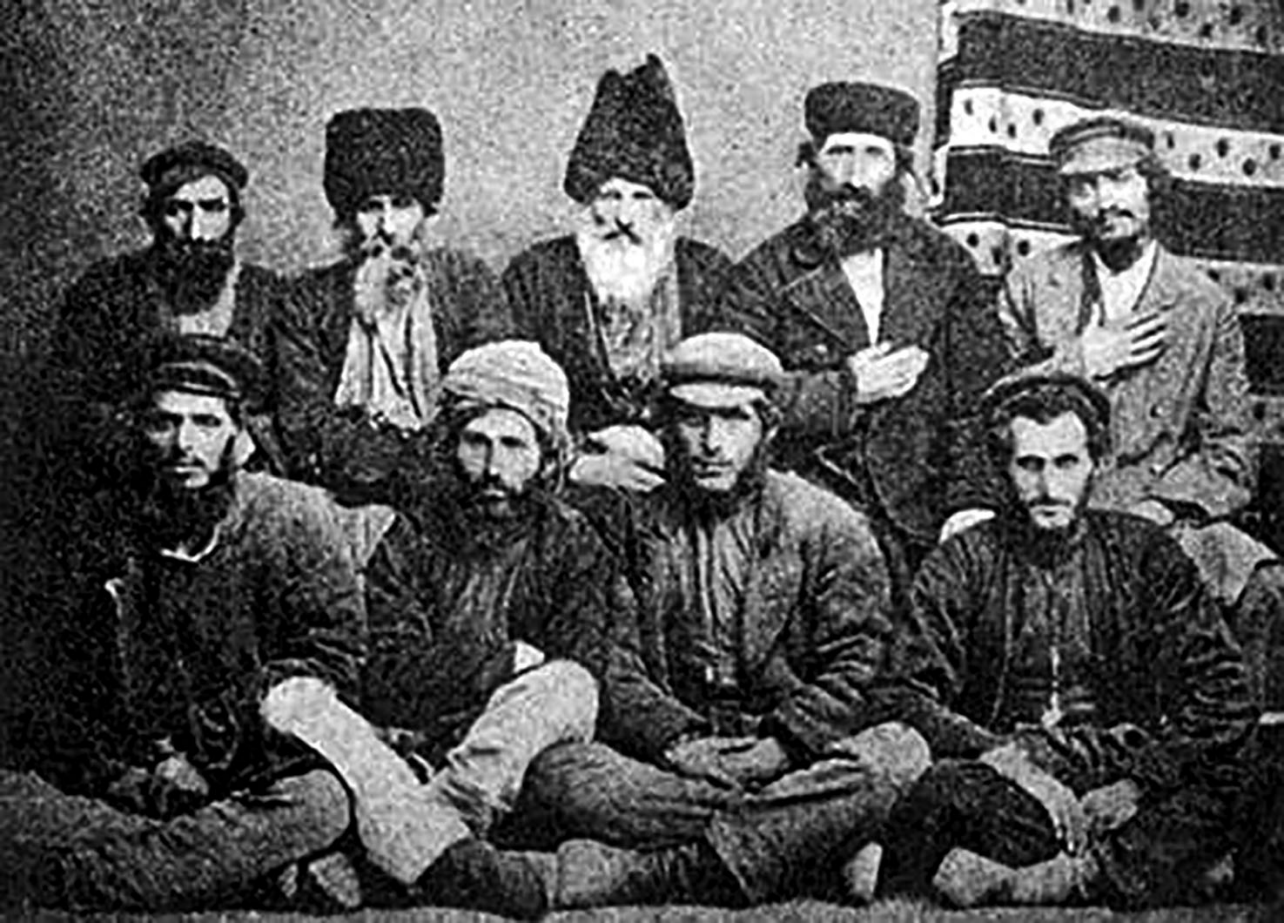
The nine defendants in civilian clothes, *c.*1879. *Source:* (see note 83).

Eleven months after Sarra Modebadze’s death, in early March 1879, nine Jewish men stood trial at the Kutaisi Circuit Court (Figure 3).^83^

Numerous local, regional and central newspapers reported from the courtroom, including *Kavkaz* (Caucasus) and the *Tiflisskii vestnik* (Tiflis Gazette), the Georgian-language *Droeba* (Times), the Russian- and Hebrew-language Jewish papers *Razsvet* from St Petersburg and *Ha-Melitz* from Odessa, and even the *Pravitel’stvennyi vestnik* (Government Gazette).^84^ Some of them had sent their own stenographers to report directly to their readers.^85^ In the liberal post-reform climate, testimony and speeches in court were not subject to censorship and were usually printed in full. To be able to accommodate the full transcript of the ten-day trial, *Kavkaz* devoted one of its four pages to the Kutaisi case for a total of six weeks. According to the *Tiflisskii vestnik*, public interest was enormous, but since the courtroom was small, no more than 100 tickets could be made available for spectators.^86^ It was the Beilis trial of its time, albeit on a less international scale.

At the same time, the Georgian journalist Sergei Meskhi reported with consternation from Kutaisi that the belief in Jews kidnapping Christian children for their blood was firmly entrenched, not only among ‘rural, uneducated, and superstitious people’, but also among ‘many educated’ ones.^87^ Another local conviction, he added, was that the outcome of the trial was a done deal: the Christians of Kutaisi were certain that Jewish financial resources would ensure an acquittal.^88^ Clearly, antisemitism had become a powerful force, and blood libel was only one aspect of it.

Still, the state prosecution was careful with the wording, and the formal charge was abduction resulting in death, not ritual murder. The implications were clear though:

I accuse the defendants present here: residents of the village of Sachkhere in Shorapani District—Iskhak, Shamuel, Bichia and Mordakh Khundiashvili as well as Iskhak, Moshe and Iakov Tsveniashvili—of the following: that on 4 April 1878 […], they grabbed (*zakhvatili*) the young girl Sarra Modebadze, put her into a saddle bag, […] and took her to the village of Sachkhere in this manner, where she was illegally held captive […], which resulted in her death; I accuse Moshe Sotsiashvili of trying to cover up the crime and […] dump the body during the night of the Jewish Passover and Mikhail Elikishvili of knowing that his neighbours and relatives had abducted Sarra […].^89^

The curious mentioning of Passover was a wink and a nod to the blood accusation, and virtually all newspapers framed the trial in such terms. On 7 March 1879, the *Tiflisskii vestnik*, for example, left no doubts on its front page: ‘The gist of the case is the accusation that Jews kidnapped a Christian girl to extract blood, which is allegedly needed for certain rituals.’^90^

This framing accounted for the extensive press coverage of the trial and its transregional support network. Not only did local rabbis write to Professor Khvol’son, but they also mobilized Aaron Eligulashvili, one of the most influential Jewish merchants in Kutaisi, who travelled to St Petersburg and secured support from the Jewish politician and philanthropist Goratsii Gintsburg (Megrelishvili 1978a, appx). Ultimately, it was Gintsburg who approached the liberal defence lawyer Petr Aleksandrov (who had only just persuaded a jury to acquit the Russian revolutionary Vera Zasulich in 1878), while Eligulashvili hired Lev Kupernik as a second, experienced (and Jewish) lawyer from the capital. The local jurists Kikodze and Lolua completed the defence team.

The assistant prosecutor became more direct on the second day of the trial:

The defence will say: the purpose of the abduction has not been proven. Well, I can tell you that if the purpose had been proven, the charge would be very different. […] Those who expect a clarification of the religious question won’t get it in this case […] because the investigation could not prove how it is related.^91^

The ‘religious question’, namely the blood accusation, was clearly raised, and the defence knew how to read these references. Aleksandrov therefore responded:

The purpose of the abduction has not been proven, says the prosecutor, and meanwhile, twice in the same indictment, he dates different circumstances to the eve of Passover. […] What is the Jewish calendar doing in a Russian indictment bill if, as in the present case, this calendar is unrelated to the purpose of the crime?^92^

Aleksandrov thus exposed the prosecutor for trying to bring in blood libel through the back door.

The prosecutors’ case was weak, as witnesses contradicted each other. But, rather than address this blatantly illegal behaviour, perjury, they just put it down to exhaustion and ignorance and told the judges that the verdict was ‘a matter of conscience’.^93^ Aleksandrov therefore pursued a double strategy. In a speech that lasted several hours, he exposed the unlawfulness of state­ments for which people had clearly perjured themselves; at the same time, he stressed the importance of ‘justice’:

[The accused] instinctively felt that there is justice [*spravedlivost’*] and truth on earth, that their innocence must be revealed, that if the people determining their fate do not see or do not want to see that truth, then they only need to rise higher.^94^

Here, justice was invoked as a moral compass, a driving force for Jewish perseverance. It convinced them that they had to right a moral wrong. But Aleksandrov also tied the notion of ‘justice’ closely into the new court system: ‘This first public case on charges of this nature will remind the Russian people of justice [*spravedlivost’*], justice alone, which is needed so that such sad cases do not happen again.’^95^ He scolded the local investigator and prosecutor’s office for their poor work that had little to do with the ‘rule of law’:

This case will show Russian court investigators that they should not get carried away by superstition but stand above it, not succumb to perjury and slander but be critical of the facts and review them, for which the law provides them with all means.^96^

**Figure 4.**
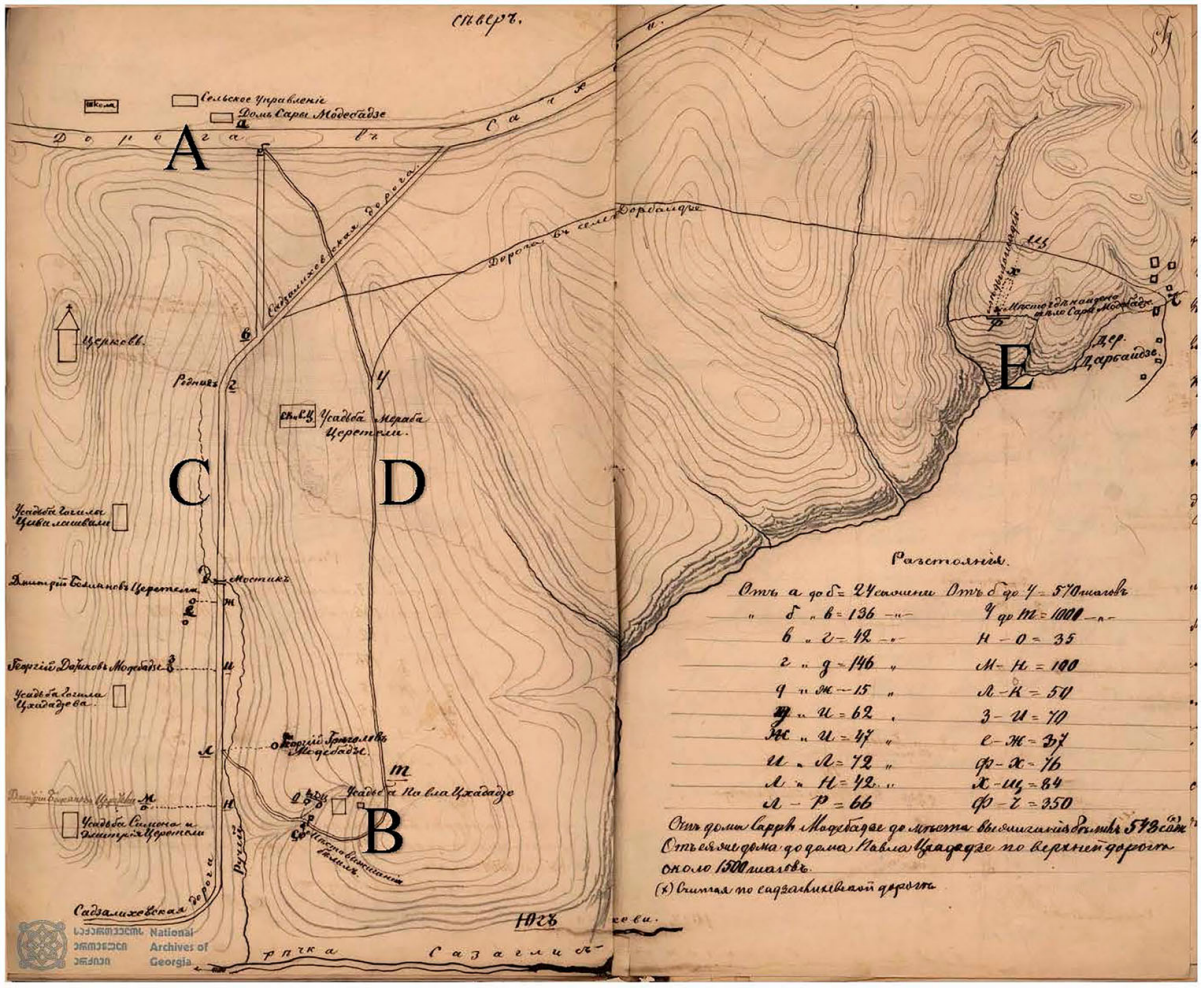
Topographical map indicating all major locations mentioned in the trial as well elevations, rivers and roads. *Source:* National Archive of Georgia (NAG), f. 7, op. 3, d. 1774, ll. 53*ob–*54. Note: The map shows the house of the Modebadze family (A), the place where Sarra had helped their neighbours and was last seen (B), the valley road through Perevisi, which would have involved an initial descent and then steep ascent for Sarra going home (C), the slightly longer but easier path across the plateau (D), and the place where her body was found (E).

To prove the unlawfulness of testimony, Aleksandrov relied on various forms of expertise, including medical and geographical: he used a topographical map of the location in question, drawn up by the Kutaisi court and showing distances and elevation (Figure 4), combined with credible testimony on where and when the girl had last been seen alive. Thus, he was able to show that Sarra Modebadze would have never even come near the Jewish men, and instead had taken the easier, upper path across the plateau to go home (where she got lost in the heavy fog, took a wrong turn, and tragically died from the cold and exhaustion).^97^

Aleksandrov also used a doctor’s calculation that the girl, if she had really been stuffed into a horse’s saddle bag, would have suffered constant friction and around 15,000 jolts and jerks during her two-hour journey to Sachkhere, which would have left countless marks on the body; unscathed as it was, the doctor concluded, the body could have never been transported this way.^98^

Faced with the defence’s piercing questions, the schoolteacher Mikadze admitted under oath that he had lied during the investigation for fear of his fellow villagers: ‘All of us who did not agree with them were hunted down and called *Yids*.’^99^

Religious experts were not summoned in this trial, not least because the prosecution had not made the blood accusation a formal part of the indictment. Still, Aleksandrov cited and praised Khvol’son’s book (1861) as convincing evidence against blood libel.^100^ Aware that the Russian publicist Ippolit Lutostanskii had just produced a widely circulated anti-Jewish diatribe defending the blood accusation, Aleksandrov preventively discarded it as a ‘mutilated and perverted reprint’ of an earlier piece and easily refuted by Khvol’son’s conscientious scholarly work.^101^ Thus, the defence made indirect use of the expert’s opinion to highlight the absurdity of the accusation.

To some observers, this approach made little sense. Since the indictment had omitted the blood accusation, some local journalists called the closing speeches by Aleksandrov and especially Kupernik, who took the time to discuss Lutostanskii chapter by chapter, a case of ‘using a sledgehammer to crack a nut’ (*streliat’ iz pushki po vorob’iam*).^102^ Notably though, the defence’s approach was rhetorically refined, meticulous, and patronizing towards the state prosecutors—and thus, it worked. The court did not take long to deliberate and acquit all defendants. The verdict was met with sustained applause in the courtroom.^103^

Naturally, conservatives were fuming. Two weeks after the verdict, Dostoevsky denounced Aleksandrov as a ‘remarkable scoundrel’ (*zamechatel’nyi negodiai*) in a letter to the Russian nationalist writer Olga Novikova, before adding, ‘How disgusting that the Kutaisi *Yids* were acquitted. They are undoubtedly guilty’ (Dostoevsky 1988 [1879], 59).

Be that as it may, the prosecution had tried to push a weak case. However, it was not quite an accomplice to a crime. Unlike in the Beilis case, it did not fabricate evidence or tamper with witnesses. Prosecutor Lidov, however, would not acknowledge the weakness of his argument, even after the verdict, and lodged an appeal with the Tiflis Judicial Chamber.^104^ This time, not even the central prosecutor would play this game anymore.

The full transcripts of the hearing on 29 April 1880, immediately published by the Jewish weekly *Razsvet*, reveal the reasoning of the Chamber’s prosecutor, Bykov. First, he concluded that the appeal could not present any new evidence that the little girl had ever used the main road.^105^ Second, unlike the Kutaisi prosecutor, he made the link to the blood accusation explicit, if only to rock the foundation of the case. The local Christians could not simply mistake the cries of a little goat for the cries of an abducted child, he reasoned: ‘For these people to think in such a way, they would have to be, at least, absolutely convinced that there is a religious rite among Jews that requires the use of Christian blood.’^106^ This very fact, however, disqualified them: ‘You cannot rely on the words of people who testify […] under the impression of the prejudice that Jews make use of Christian blood.’^107^ Thus, with no evidence other than that tainted by antisemitic prejudice, the case had no lawful basis, and Bykov refused to press charges.^108^

This comforting outcome, however, leaves the question unanswered as to why the blood accusation was so entrenched to begin with. What led the local Christians to provide false testimony? I would suggest three main reasons. The first is the Surami precedent, which notably resulted in a (false) conviction and led to numerous anti-Jewish riots over the following decades. These domino effects were closely tied into the second reason: the centrality of hearsay as a form of communication. Rumours were the ‘fake news’ of old, and bad rumours in particular travelled fast.

Third, there was colonialism and local economic prejudice. Far fewer officials stirred antisemitic sentiment than in Surami and other cases, and thus it was rather the economic and legal situation in Kutaisi province, as a result of colonial rule, that could be seen as contributing factors. The Surami precedent itself was a colonial construct. So was the economic context: many Georgian Jews were released from serfdom from 1865 and remained in complete dependence upon their former landlords. In Sachkhere, many had been serfs on the estate of the landowner Prince Tsereteli (Mamistvalishvili 2014, 120–121). Without access to land of their own, their only choice for making a living after emancipation was trade, which made them vulnerable to accusations of ‘exploiting’ the Christians. According to the journalist Meskhi, it was a ‘social and economic rather than religious hatred’ that people felt towards the Jews.^109^ The only solution was to grant them full rights, he argued, so that they would ‘abandon the desire to deceive and oppress Christians’.^110^ Meskhi and other educated local observers thus clearly harboured antisemitic prejudice—and yet still did not support the blood accusation. He was one of many Georgian journalists who dismissed it as a sad superstition that had nothing to do with Judaism and which had been refuted by experts countless times.^111^ The rural population, however, cared less about expert opinion and was more susceptible to the toxic mixture of economic prejudice, rumours and established religious superstition.

## Conclusions

The Kutaisi Trial was a milestone. For the first time in the history of blood libel in Russia, the accused were acquitted. If we compare the case with earlier and later ones—such as Velizh, Saratov and Surami, on the one hand, and Beilis, on the other—it takes up a curious interim position. The ‘tools of modernity’ (not least, international publicity) were not yet as refined as they would later become. The trial was closely observed by newspapers in and beyond the region, especially Jewish ones (such as *Razsvet*/St Petersburg, *Ha-Melitz*/Odessa, *Ha-Tsefirah*/Warsaw, and *Ha-Magid*/Lyck, East Prussia). The *Ha-Melitz* editors and publishing house, in fact, produced the most detailed of all available transcripts of the trial.^112^ And yet, Kutaisi never became a truly global affair—certainly not when compared with the Beilis case. Further, while the defence drew on scientific expertise, the prosecution mostly relied on (false) testimony, which was similar to many pre-modern cases. At the same time, Kutaisi shows a public trial and a highly active and differentiated judiciary in which talented Russian defence lawyers did not hesitate to humiliate state institutions. The local and central prosecutions did not have a shared agenda, either. Clear differences also emerged between local and central representatives of state power. That said, differences of opinion were not the key novelty. The novelty lay in the institutions themselves: in their visibility and public accessibility, and in the role that ‘justice’ and ‘lawfulness’, as discursive and practical strategies, would play in them. Not only the jurists but also the Jewish petitioners used these strategies, consistently highlighting the legality and illegality of actions.

What about the role of the Russian administration and, by extension, colonialism beyond economic factors? By the second half of the nineteenth century, it was not only the political and legal spheres that had been colonized (with Russian governors and law enforcement agencies), but the cultural sphere, as well; Russian had become the language of state institutions and the dominant language of the press and growing ‘public sphere’ in Tiflis. As elsewhere in the empire, key figures of the Russian Orthodox Church joined forces with Russian conservatives and nationalists, which contributed to a surge in antisemitic rhetoric. Russia had exported antisemitism, one is tempted to conclude, not least because no comparable cases of blood libel had occurred in Georgia prior to Russian colonization.

That said, the Kutaisi case certainly shows Russian actors in a variety of roles. When local accusations of blood libel emerged, a Russian governor, court investigator, and regional prosecutor happily jumped on the bandwagon—perhaps to further their careers, perhaps because they actually believed in the blood accusation, or simply out of antisemitism. And while they were not the driving force behind the accusation, they did everything in their power to keep it alive. In so doing, however, and unlike in previous cases, they met with formidable Russian opposition: from the defence lawyers and the prosecutors of the Judicial Chamber, and from the viceroy and his administration. While the former two were mostly interested in ‘legality’ and ‘justice’, the latter cared more about stability (and may have preferred the case to go away as quickly as possible). In addition, most of the Tiflis-based (Russian-language) newspapers highlighted the absurdity of the accusation, none of the doctors (all Russians) spoke ‘in favour’ of the prosecution, and the acquittal was met with jubilation in the courtroom. Kutaisi was a far cry from Surami.

Undoubtedly, Russian colonialism had strengthened antisemitism in Georgia in the mid-nineteenth century. However, far from being a solely ‘Russian phenomenon’, this antisemitism was shared across national and social groups. More importantly, by the late 1870s, the same colonialism had delivered some of the tools to fight antisemitism, notably an independent judiciary, in which not even the central prosecution would play along, and a diverse press. It had also created ever more connections for the Georgian Jews to Ashkenazi Jews elsewhere in the empire, whose help proved crucial to winning the case.

Were ‘the power of Russian liberalism’ and ‘the people’s war against tyranny’, to quote Megrelishvili, more likely to shine in peripheral courts? Perhaps Russian liberals would see particular opportunities and good causes there, defending the empire’s internal ‘others’? Not necessarily. While there is evidence that post-reform jurists vigilantly defended the rights, for example, of Muslims in Crimea and Kazan (Kirmse 2019), most jurists preferred more fashionable, central locations. Judges, prosecutors, and defence lawyers were generally in short supply in the borderlands (Baberowski 1996, 385–403; Kirmse 2019, 136–137). And while the lawyer Aleksandrov defended the Sachkhere Jews with great passion, he had defended the Russian revolutionary Vera Zasulich just as passionately the previous year.

Either way, the acquittal did not put an end to local antagonism. Seen as predetermined and illegitimate, it was soon followed by renewed assaults.^113^ These attacks even intensified after the appeal case fell apart the following spring, with *Droeba* featuring various articles on anti-Jewish attacks, robberies, and humiliations between 1880 and 1884.^114^ And yet, in the six years after the trial, the Jewish population would still double in Kutaisi province, from 3516 in 1880 to 7082 in 1886.^115^ To some, the acquittal was evidence that Kutaisi was a safe place for Jews—and may have played a role in this influx. Kutaisi city, in particular, offered not only the protection of its sizable Jewish community but also increasing economic opportunities. Either way, the growth also underlined that, however difficult and traumatic the experience of blood libel might have been (and continued to be), the Jewish population had no intention of leaving.

## Notes

1 The archive in Kutaisi holds no record of this trial. However, the documents at the National Archive in Tbilisi (see note 15) are rather varied and include court and administrative records and correspondence, covering a wide range of actors in both Kutaisi and Tbilisi.

2 The Multan case of 1892 is a notable exception. It took place in the Volga-Kama region, and the people charged with ritual murder were Votiaks—an ethnic group that spoke a Finnic language and, at the time, practiced religious syncretism that included animist rituals. After almost five years of trials, with two ‘guilty’ verdicts that were both annulled by the Governing Senate, they were acquitted at last in 1896. As it turned out later, Russian peasants had committed this murder and made it look like a ritual killing (Geraci 2002, 195–222).

3 On the multiple meanings of ‘justice’ in late imperial Russia, see Pecherskaya (2005), Kuße and Plotnikov (2011) and Lenkewitz-Salminen (2019).

4 For a direct comparison, see Lindemann (1991). Beilis himself reported that, once in custody, fellow prisoners told him he was ‘a second Dreyfus’, attesting to the popularity of this discourse even among ordinary people (Levin 2014, 107–108). At the end of the Beilis trial, the prosecution referred to the Dreyfus case as an example of a case that had mainly caused concern around the world because the defendant was Jewish; *Delo Beilisa. Stenograficheskii otchet*, Vol. 3 (Kiev, 1913), 4.

5 See note 2.

6 They all base their analyses on the correspondence between Vorontsov and Montefiori, first published by a Jewish newspaper in Odessa: “Perepiska kn. M. Vorontsova s M. Montefiore (po delu ob obvinenii evreev g. Surami v ubienii khristianskogo mal’chika)”. *Den’*, 31 January 1871, no. 5, pp. 75–76. Soon it was reprinted in Georgia as “Perepiska kn. M. Vorontsova s M. Montefiore”. *Kavkaz*, 19 February 1871, no. 22, pp. 2–3.

7 See the article by Yulia Oreshina in this special issue.

8 See ‘Perepiska’ (1871) and Loewe (1890), 2: 23.

9 See note 6.

10 *Ha-Magid* [The Narrator], “Gurjistan (gruzia)”, 25 August 1869, no. 33: 259–260.

11 In addition to those already mentioned, see Tager (1933) and Rogger (1986) on Beilis.

12 It was published in 1879 in three different forms: as A. E. Landau (ed.), *Kutaisskii protsess: delo o pokhishchenii i umervshchlenii evreiami krestianskoi devochki Sarry Iosifovoi Modebadze* (St Petersburg: Tipo-Litografiia Landau); as A. E. Landau (ed.), `Kutaisskoe delo'. In *Evreiskaia biblioteka. Istoriko-literaturnyi sbornik*, Vol. VII (St Petersburg: Tipo-Litografiia Landau); and as *Kutaisskoe delo: polnyi stenograficheskii otchet o Kutaisskom dele s prilozheniem resheniia suda v okonchatel'noi forme i topograficheskoi s'emki mestnosti* (St Petersburg: Izdanie redaktora gazety ‘Hamelitz’).

13 Kavkazskii statisticheskii komitet, *Sbornik svedenii o Kavkaze*. N. Seidlitz (Tiflis, 1880), 7: xxxvii–xxxviii; https://viewer.rsl.ru/ru/rsl01003861881?page=586&rotate=0&theme=white/.

14 The basic facts are summarized in the indictment bill; *Kutaisskoe delo: polnyi stenograficheskii otchet*, 3–15.

15 National Archive of Georgia (NAG), Central Historical Archive, Tbilisi, F. 7 (Main Department of the High Commissioner of the Civil Administration of the Caucasus), op. 3 (judicial department), d. 1774 (‘Complaint by representatives of the Jewish community in Sachkhere village’, 1878–79), ll. 10–10*ob*.

16 *Kutaisskoe delo: polnyi stenograficheskii otchet*, 196 (Darbaidze’s testimony), 226 (Abashidze’s testimony).

17 “Samartali. uriebis sakme [Law. Yids’ Case]”. *Droeba*, 10 March 1879, no. 53: 3. The official transcripts (in Russian) do not contain this exact passage. They confirm that the father indeed started believing in an abduction once he had heard about Jews passing through the village, but suggest that he only admitted his awareness of Surami when asked by the defence if he had heard about the case. Still, as the father testified in Georgian, the *Droeba* stenographer may have captured the statement more accurately than the court stenographer; *Kutaisskoe delo: polnyi stenograficheskii otchet*, 18, 24.

18 *Kutaisskoe delo: polnyi stenograficheskii otchet*, 226; NAG, F. 7, op. 3, d. 1774, l. 10*ob*.

19 According to the Statutes of Criminal Court Procedure, ‘any occurrence that bears the marks of a crime or misdemeanor shall be reported by the police immediately and no later than twenty-four hours after learning of it to the responsible court investigator and prosecution’: “Vysochaishe utverzhdennyi Ustav ugolovnago sudoproizvodstva (Statutes of Criminal Court Procedure)”, in *Polnoe sobranie zakonov Rossiiskoi imperii, sobranie vtoroe* (PSZ II), vol. 39, pt 2, no. 41476, 20 November 1864, art. 250.

20 *Kutaisskoe delo: polnyi stenograficheskii otchet*, 5.

21 NAG, F. 7, op. 3, d. 1774, l. 5*ob*.

22 *Kutaisskoe delo: polnyi stenograficheskii otchet*, 6.

23 Ibid.

24 *Kutaisskoe delo: polnyi stenograficheskii otchet*, 5.

25 Ibid.

26 NAG, F. 7, op. 3, d. 1774, l. 11.

27 NAG, F. 7, op. 3, d. 1774, ll. 1–1*ob*.

28 NAG, F. 7, op. 3, d. 1774, l. 1*ob*.

29 NAG, F. 7, op. 3, d. 1774, l. 2.

30 NAG, F. 7, op. 3, d. 1774, l. 5 (dated 15 May 1878).

31 NAG, F. 7, op. 3, d. 1774, l. 2.

32 Ibid.

33 NAG, F. 7, op. 3, d. 1774, ll. 2 (petitioners’ claim), 15*ob*. (report by Head of Viceroy’s Administration).

34 NAG, F. 7, op. 3, d. 1774, l. 5*ob*.

35 NAG, F. 7, op. 3, d. 1774, l. 10*ob*.

36 NAG, F. 7, op. 3, d. 1774, l. 12.

37 Ibid.

38 NAG, F. 7, op. 3, d. 1774, ll. 12*ob*., 35*ob*–36.

39 NAG, F. 7, op. 3, d. 1774, ll. 10*ob*.–11, 38*ob*–39.

40 NAG, F. 7, op. 3, d. 1774, l. 5*ob*.

41 NAG, F. 7, op. 3, d. 1774, ll. 12*ob*, 35*ob*.

42 NAG, F. 7, op. 3, d. 1774, ll. 12–12*ob*.

43 *Kutaisskoe delo: polnyi stenograficheskii otchet*, 6.

44 NAG, F. 7, op. 3, d. 1774, l. 12.*ob*.

45 NAG, F. 7, op. 3, d. 1774, l. 13.

46 Ibid.

47 NAG, F. 7, op. 3, d. 1774, l. 2*ob*.

48 Ibid.

49 Ibid.

50 NAG, F. 7, op. 3, d. 1774, l. 3*ob*.

51 “Blood libels against Jews in Modern Times. 1600–1965”, in *Encyclopaedia Judaica*, vol. 4 (1971): 1128–1131 (‘In Russia’), esp. 1129.

52 NAG, F. 7, op. 3, d. 1774, l. 4.

53 NAG, F. 7, op. 3, d. 1774, l. 6.

54 NAG, F. 7, op. 3, d. 1774, ll. 21–22*ob*.

55 NAG, F. 7, op. 3, d. 1774, l. 21*ob*.

56 Ibid.

57 NAG, F. 7, op. 3, d. 1774, l. 22*ob*.

58 NAG, F. 7, op. 3, d. 1774, ll. 22, 22*ob*.

59 NAG, F. 7, op. 3, d. 1774, ll. 24–24*ob* (dated 24 June).

60 On this person’s illustrious career, see “Andreev, Valerian Alekseevich”, in *Al’manakh sovremennykh russkikh gosudarstvennykh deiatelei* (St Petersburg: Tip. Isidora Gol’dberga, 1897), 205–206.

61 NAG, F. 7, op. 3, d. 1774, l. 15*ob*.

62 NAG, F. 7, op. 3, d. 1774, l. 16.

63 Ibid.

64 NAG, F. 7, op. 3, d. 1774, ll. 16*ob*–17.

65 NAG, F. 7, op. 3, d. 1774, l. 17*ob*.

66 Ibid.

67 NAG, F. 7, op. 3, d. 1774, ll. 26, 28*ob*, 29.

68 NAG, F. 7, op. 3, d. 1774, l. 30.

69 NAG, F. 7, op. 3, d. 1774, ll. 46–53, esp. 52*ob*.

70 NAG, F. 7, op. 3, d. 1774, ll. 31*ob*–32.

71 NAG, F. 7, op. 3, d. 1774, l. 33*ob*.

72 NAG, F. 7, op. 3, d. 1774, ll. 26*ob*–27*ob*.

73 NAG, F. 7, op. 3, d. 1774, ll. 28–28*ob*.

74 NAG, F. 7, op. 3, d. 1774, l. 25.

75 NAG, F. 7, op. 3, d. 1774, ll. 59–59*ob*.

76 NAG, F. 7, op. 3, d. 1774, l. 60.

77 NAG, F. 7, op. 3, d. 1774, l. 58*ob*.

78 NAG, F. 7, op. 3, d. 1774, l. 58.

79 NAG, F. 7, op. 3, d. 1774, ll. 63–63*ob*.

80 NAG, F. 7, op. 3, d. 1774, ll. 71–71*ob*.

81 NAG, F. 7, op. 3, d. 1774, ll. 73*ob*–74.

82 NAG, F. 7, op. 3, d. 1774, l. 74.

83 The origins of the photograph remain unclear. It is neither part of the archival records in Tbilisi nor included in the original publications of the trial transcripts. It appeared in the Hebrew translation of the court proceedings (Megrelishvili 1978a), albeit in poorer resolution than presented here, with the editor noting that he had received the image from a certain Gershon Tsitsiashvili. In all likelihood, it was taken after the initial acquittal in 1879; people in custody or court in the Russian Empire usually wore prisoners’ clothes. Since one of the accused died before the appeal in 1880 (mentioned in “Delo Sachkherskikh evreev”, *Razsvet*, no. 20, 15 May 1880: 798), the photograph was probably taken shortly after the first acquittal. It now circulates freely on the internet; e.g., http://callofzion.ru/pages.php?cid=21&start=8; http://jewishfreedom.org/page770.html/.

84 Neither Hebrew nor Georgian use capitalization. For reasons of greater consistency in lists and enumerations, however, I have decided to capitalize the first letter when transliterating names and publication titles into Latin script.

85 “Mestnye izvestiia: Kutais, 8 marta”. *Kavkaz*, no. 56, 11 March 1879: 2.

86 “Kutais, 5-go marta”. *Tiflisskii vestnik*, no. 35, 7 March 1879: 3.

87 Sololak’eli (pseudonym for Sergei Meskhi), “Maslaati [Discussion]”. *Droeba*, 18 March 1879, no. 60: 2.

88 Ibid.

89 *Kutaisskoe delo: polnyi stenograficheskii otchet*, esp. 310–311.

90 “Tiflis, 5-go marta”. *Tiflisskii vestnik*, no. 35, 7 March 1879: 1.

91 *Kutaisskoe delo: polnyi stenograficheskii otchet*, 318.

92 *Kutaisskoe delo: polnyi stenograficheskii otchet*, 369–370.

93 *Kutaisskoe delo: polnyi stenograficheskii otchet*, 310, 316.

94 *Kutaisskoe delo: polnyi stenograficheskii otchet*, 320–321.

95 *Kutaisskoe delo: polnyi stenograficheskii otchet*, 385.

96 Ibid.

97 *Kutaisskoe delo: polnyi stenograficheskii otchet*, 361–365.

98 *Kutaisskoe delo: polnyi stenograficheskii otchet*, esp. 304–305, 324.

99 “Mestnye izvestiia: Kutais, 8 marta”. *Kavkaz*, no. 56, 11 March 1879: 3; see also “Zabytaia cherta evreiskago dela. Okonchanie”. *Kavkaz*, no. 88, 22 April 1879: 1.

100 *Kutaisskoe delo: polnyi stenograficheskii otchet*, 371.

101 *Kutaisskoe delo: polnyi stenograficheskii otchet*, 371–372.

102 “Zabytaia cherta evreiskago dela”, 2.

103 *Kutaisskoe delo: polnyi stenograficheskii otchet*, 418.

104 “Delo Sachkherskikh evreev”. *Razsvet*, no. 20, 15 May 1880: 794.

105 *Razsvet*, nos 20–26, 15 May–26 June 1880. The transcripts were later reprinted in Hebrew (Megrelishvili 1978a) and German (Megrelishvili 1978b).

106 “Delo Sachkherskikh evreev”. *Razsvet*, no. 21, 22 May 1880: 836.

107 Ibid., 836, 837.

108 “Telegramma”. *Razsvet* no. 18, 1 May 1880: 684.

109 Sergei Meskhi, “Uriebis sdevna imeretshi [Persecution of *Yids* in Imereti]”. *Droeba*, 29 June 1879, no. 135: 2.

110 Sergei Meskhi, “Ebraelebis shesakheb [Concerning the Jews]”. *Droeba*, 19 January 1875, no. 9: 1.

111 For example: “Dghiuri [Daily news]”. *Droeba*, 17 March 1876, no. 28: 1; “Dghiuri”. *Droeba*, 29 May 1877, no. 59: 2; I. Alkhazashvili, “4 ap’rilis ambavi suramshi [The Story of 4 April in Surami]”. *Droeba*, 27 April 1877, no. 46: 2–3; “Dghiuri”. *Droeba*, 4 June 1878, no. 108: 2; Sergei Meskhi, “Uriebis sakme [Yids’ Case]”. *Droeba*, 21 February 1879, no. 38: 1.

112 See note 12.

113 Meskhi, “Uriebis sdevna imeretshi”, 2; M. B. Jvarisubneli (pseudonym), “ts’erili redakt’ortan [Letter to the editor]”. *Droeba*, 1 July 1879, no. 136: 4.

114 Vinme Israeli [Some Jew], “Droebis k’oresp’ondentsia, Kareli [Droeba Correspondent, Kareli]”. *Droeba*, 9 August 1881, no. 165: 2; “Dghiuri [Daily News]”. *Droeba*, 23 June 1882, no. 129: 2; A. Akopovi, “Chveneburs uriebzed [About Our Yids]”. *Droeba*, 25 July 1882, no. 153: 1–2; “Shinauri kronik’a [Domestic Chronicle]”. *Droeba*, 4 October 1883, no. 195: 2; “Shinauri kronik’a [Domestic Chronicle]”. *Droeba*, 24 March 1884, no. 66: 2–3. There were also further Jewish petitions to the viceroy, e.g., NAG, F. 7, op. 3, d. 1774, ll. 75–77; see also ll. 66–67.

115 Cf. *Sbornik svedenii o Kavkaze*. P. XXXVIII (1880), and the following map with statistical data from 1886: https://upload.wikimedia.org/wikipedia/commons/9/94/Map-etno-kutais.jpg/.
